# Chronic kidney disease and cause-specific hospitalisation: a matched cohort study using primary and secondary care patient data

**DOI:** 10.3399/bjgp18X697973

**Published:** 2018-07-17

**Authors:** Masao Iwagami, Ben Caplin, Liam Smeeth, Laurie A Tomlinson, Dorothea Nitsch

**Affiliations:** Department of Non-communicable Disease Epidemiology, London School of Hygiene and Tropical Medicine, London.; Centre for Nephrology, University College London, London.; Department of Non-communicable Disease Epidemiology, London School of Hygiene and Tropical Medicine, London.; Department of Non-communicable Disease Epidemiology, London School of Hygiene and Tropical Medicine, London.; Department of Non-communicable Disease Epidemiology, London School of Hygiene and Tropical Medicine, London.

**Keywords:** acute kidney injury, chronic kidney diseases, general practice, heart failure, hospitalisation, infection

## Abstract

**Background:**

Although chronic kidney disease (CKD) is associated with various outcomes, the burden of each condition for hospital admission is unknown.

**Aim:**

To quantify the association between CKD and cause-specific hospitalisation.

**Design and setting:**

A matched cohort study in primary care using Clinical Practice Research Datalink linked to Hospital Episode Statistics in England.

**Method:**

Patients with CKD (estimated glomerular filtration rate <60 mL/min/1.73 m^2^ for ≥3 months) and a comparison group of patients without known CKD (matched for age, sex, GP, and calendar time) were identified, 2004–2014. Outcomes were hospitalisations with 10 common conditions as the primary admission diagnosis: heart failure; urinary tract infection; pneumonia; acute kidney injury (AKI); myocardial infarction; cerebral infarction; gastrointestinal bleeding; hip fracture; venous thromboembolism; and intracranial bleeding. A difference in the incidence rate of first hospitalisation for each condition was estimated between matched patients with and without CKD. Multivariable Cox regression was used to estimate a relative risk for each outcome.

**Results:**

In a cohort of 242 349 pairs of patients, with and without CKD, the rate difference was largest for heart failure at 6.6/1000 person-years (9.7/1000 versus 3.1/1000 person-years in patients with and without CKD, respectively), followed by urinary tract infection at 5.2, pneumonia at 4.4, and AKI at 4.1/1000 person-years. The relative risk was highest for AKI with a fully adjusted hazard ratio of 4.90, 95% confidence interval (CI) = 4.47 to 5.38, followed by heart failure with 1.66, 95% CI = 1.59 to 1.75.

**Conclusion:**

Hospitalisations for heart failure, infection, and AKI showed strong associations with CKD in absolute and(or) relative terms, suggesting targets for improved preventive care.

## INTRODUCTION

Chronic kidney disease (CKD) is a common condition in the community.^1^^,^^2^ In the UK, according to the Quality and Outcomes Framework,^3^ GPs have been incentivised to register patients with CKD stages 3–5 (estimated glomerular filtration rate [eGFR] <60 mL/min/1.73 m^2^ for ≥3 months) since 2006. The majority of these patients are in CKD stage 3 (eGFR of 30–59 mL/min/1.73 m^2^) and managed by GPs without referral to nephrology services.^4^

There have been concerns, particularly among GPs, regarding potential overdiagnosis of CKD in older patients with mildly reduced kidney function.^5^^,^^6^ Some have argued that labelling people as having CKD may unnecessarily create anxiety, while only a minority of patients with CKD progress to end-stage kidney disease requiring renal replacement therapy.^7^^,^^8^ However, patients with CKD are at higher risk of cardiovascular events, death, and all-cause hospitalisation,^9^ an important outcome for both patients and the national healthcare system.^10^ Awareness of the causes of admission among patients with CKD could offer opportunities for prevention. However, previous studies suggesting a positive association between CKD and hospitalisations have not investigated the specific causes in detail.^11^^–^13

Accumulating evidence suggests that CKD is causally associated with a wide range of adverse outcomes, including acute kidney injury (AKI),^14^ cardiovascular (myocardial infarction,^15^^,^^16^ heart failure,^17^^,^^18^ and stroke^19^^,^^20^) and non-cardiovascular conditions (infection,^21^^,^^22^ bleeding,^23^^,^^24^ venous thromboembolism,^25^^,^^26^ and fracture^27^^,^^28^). However, to the researchers’ knowledge, there has been no study examining the extent to which these conditions explain the increased risk of all-cause hospitalisation in patients with CKD. Identification of more common and specific causes of admission among patients with CKD is warranted to reaffirm the importance of identifying CKD in primary care and guide areas of focus for outpatient management of these patients.

Therefore, this study aimed to quantify the association between CKD and cause-specific hospitalisation, using a primary care database linked to hospital admission data. The main purpose of the study was to estimate and rank the size of absolute risk difference and relative risk between patients with and without CKD (matched for age, sex, GP, and calendar time) across 10 common causes of hospital admission.

## METHOD

### Data sources

The Clinical Practice Research Datalink (CPRD) is a database of routinely recorded primary care electronic health record data.^29^ The database represents around 7% of the UK population and includes the following information: patient demographics; coded diagnoses (Read Codes); prescriptions; laboratory test results; and referrals made by GPs. The CPRD can be linked with Hospital Episode Statistics (HES), which contains details of all hospital admissions at NHS hospitals in England and consists of main and subsidiary diagnoses, using the 10th revision of International Classification of Disease (ICD-10) codes.^30^ Currently around 400 GPs in CPRD have consented to linkage with HES, representing 75% of English practices in CPRD.^29^

**How this fits in**

Although chronic kidney disease (CKD) is associated with a wide range of adverse outcomes, more strongly associated conditions with hospital admission among patients with CKD are unknown. This study is the first to examine the association between CKD and common reasons for hospital admission in a systematic way and highlights the high burden of hospitalisation due to heart failure, infection, and acute kidney injury among patients with CKD compared with the general population. These findings suggest that, aside from prevention of end-stage kidney disease, there are important high-priority outcomes that warrant identification of CKD in primary care and improved preventive care of patients with CKD in the community.

### Study population and matched cohort

All adults in HES-linked CPRD from 1 April 2004 to 31 March 2014 were potentially eligible for inclusion. Patients were eligible for inclusion at the latest of: 1 year after practice registration,^31^ the date that the GP reached CPRD quality standards,^29^ and 1 April 2004. Patients already on renal replacement therapy (haemodialysis, peritoneal dialysis, or kidney transplantation) at cohort entry were excluded.

First, patients with CKD (stages 3–5) were identified, defined as two consecutive measurements of eGFR <60 ml/min/1.73 m^2^ for ≥3 months.^32^ Estimated GFR was calculated from serum creatinine records in CPRD (after multiplication of 0.95 to allow for lack of creatinine calibration^33^) using the Chronic Kidney Disease Epidemiology Collaboration equation.^34^ Patients, including those who had CKD before April 2004, were included in the cohort on the date when they first satisfied the CKD definition (second eGFR <60 mL/min/1.73 m^2^) after their eligibility.

Second, for a comparison group, patients without known CKD were randomly selected from the rest of the study population in a 1:1 ratio, matched for age, sex, GP, and calendar time.

### Outcomes and follow-up

The primary diagnosis in the first episode (a single period of care under one consultant team) within a spell (a patient’s entire stay in hospital) in the HES was examined; this was considered to be the main reason why a patient required hospital admission.^30^ Outcomes of this study were hospitalisations for 10 common conditions as the primary admission diagnosis: heart failure; urinary tract infection; pneumonia; AKI; myocardial infarction; cerebral infarction; gastrointestinal bleeding; hip fracture; venous thromboembolism; and intracranial bleeding, defined using ICD-10 codes (Appendix 1). In this study, the researchers focused on the first hospitalisation for each condition after cohort entry.

For each outcome, a patient was followed up until the first hospitalisation for that outcome or the end of eligibility (initiation of renal replacement therapy, death, change of GP, last data collection from the GP, or 31 March 2014), meaning that every patient could develop more than one of the outcomes. In addition, individuals selected in the comparison group (patients without known CKD) could be found to have CKD later; in this situation they were censored at the time of satisfying the CKD definition because they were already included in the CKD group from that point forward.

### Covariates

In addition to the matched factors, the researchers accounted for potential confounders in the association between CKD and cause-specific hospitalisation: ethnicity; socioeconomic status; smoking status; body mass index (BMI); and 17 comorbidities (asthma, atrial fibrillation, cancer, chronic obstructive pulmonary disease [COPD], coronary heart disease, dementia, depression, diabetes mellitus, epilepsy, heart failure, hypertension, hypothyroidism, severe mental illness, osteoporosis, peripheral arterial disease, rheumatoid arthritis, and stroke and transient ischaemic attack [TIA]).

Patients with no record of ethnicity were classed as white, based on previous studies in UK primary care.^35^^,^^36^ Socioeconomic status was assigned by quintile at an individual level using the Index of Multiple Deprivation as a composite area-level marker of deprivation.^37^ Smoking status and BMI were assigned using the data recorded closest to the cohort entry. The definitions of hypertension and diabetes were based on relevant diagnosis codes recorded before the cohort entry or prescription (antihypertensive and antidiabetic drugs, respectively) in the past 1 year prior to the cohort entry. Other comorbidities were based on relevant diagnosis codes recorded before the cohort entry.

### Statistical analysis

The baseline characteristics of matched patients with and without CKD were compared using χ^2^ tests. Incidence rates of each outcome in matched patients with and without CKD, respectively, were estimated and a difference of incidence rates between the groups was calculated. Multivariate Cox regression analyses for each outcome were then conducted, stratified by matched set to account for the matching on age, sex, GP, and calendar time (Model 1). Further adjustments were made for ethnicity, socioeconomic and smoking status, BMI, and diabetes mellitus (Model 2). Instead of excluding patients with a missing status of smoking or BMI from the analysis, an additional absent category was included for these patients to maintain the matched set between patients with and without CKD. Subsequently, adjustments for comorbidities not directly related to CKD (asthma, cancer, COPD, dementia, depression, epilepsy, hypothyroidism, severe mental illness, osteoporosis, and rheumatoid arthritis)^38^ (Model 3) were made, and also for comorbidities, which may occur concordantly with CKD (atrial fibrillation, coronary heart disease, heart failure, hypertension, peripheral arterial disease, stroke and TIA)^38^ (Model 4). A fully adjusted sub-hazard ratio for each outcome using the Fine and Gray model was estimated to account for potential competing risk (initiation of renal replacement therapy and death) between patients with and without CKD. Because of the computational burden related to size of the dataset, this competing risk analysis was conducted using a 20% random sample of the whole dataset. All the statistical analyses were carried out using STATA (version 14).

### Subgroup analyses

Several subgroup analyses were conducted. First, as previous studies suggested that the impact of CKD on outcomes may change with age,^39^^,^^40^ all the analyses were repeated by classifying the study population into two age groups; ≥75 and <75 years. Second, to examine the extent of graded association between CKD stage and cause-specific hospitalisation, the researchers conducted Cox regression analyses by dividing patients with CKD according to baseline CKD stage: 3a (eGFR 45–59 mL/min/1.73 m^2^), 3b (eGFR 30–44 mL/min/1.73 m^2^), and 4 or 5 (eGFR <30 mL/min/1.73 m^2^).^32^ Third, to see the impact of CKD on cause-specific hospitalisation among patients with no history of cardiovascular disease, patients with either diagnosis of atrial fibrillation, coronary heart disease, heart failure, peripheral arterial disease, and stroke and TIA at the cohort entry were excluded, and Cox regression analyses were performed by CKD stage. In the second and third subgroup analyses where the matched nature between patients with and without CKD was no longer maintained, adjustments for age, sex, and financial year were made, and robust standard errors to allow for clustering by GP were used, instead of stratification by matched set in the Cox regression models.

## RESULTS

**Figure 1.**
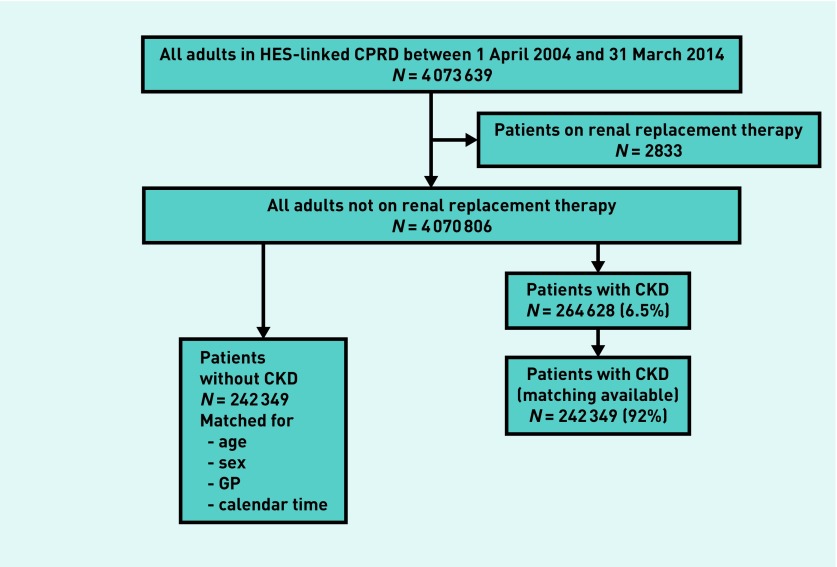
***Selection method of matched patients with and without CKD from the general population. CKD = chronic kidney disease. CPRD = Clinical Practice Research Datalink. HES = Hospital Episode Statistics.***

Among 4 070 806 eligible patients not requiring renal replacement therapy (mean age 42.7 [SD 18.8] years, male 48.8%), the researchers identified 264 628 (6.5%) patients with CKD (mean age 76.4 [SD 10.0] years, male 38.7%) (Figure 1). Of those with CKD, 242 349 (92%) were matched with patients without CKD (mean age 75.4 [SD 9.7] years, male 39.3%). Unmatched 22 279 patients with CKD (8% of those with CKD) were older and more likely to be female (mean age 87.9 [SD 5.4] years, male 31.5%). Patients with CKD were more likely to have a deprived socioeconomic status, be ex-smokers, and overweight, with a larger number of comorbidities (Table 1). Total length of follow-up, if not censored for each cause-specific hospitalisation, was 2.0 million person-years (mean 4.2 [SD 2.9] years/person).

**Table 1.** Baseline characteristics of matched patients with and without chronic kidney disease

**Characteristic**	**Patients without CKD *N*= 242 349 *n* (%)**	**Patients with CKD *N*= 242 349 *n* (%)**	***P*-value**
**Age, years**			1.000
<55	6845 (2.8)	6845 (2.8)	
55–64	23 556 (9.7)	23 556 (9.7)	
65–74	71 112 (29.3)	71 112 (29.3)	
75–84	102 594 (42.3)	102 594 (42.3)	
≥85	38 242 (15.8)	38 242 (15.8)	

**Sex (male)**	95 318 (39.3)	95 318 (39.3)	1.000

**Ethnicity**			<0.001
White/not recorded	238 533 (98.4)	238 138 (98.3)	
South Asian	1796 (0.7)	2317 (1.0)	
Black	1156 (0.5)	1060 (0.4)	
Other	864 (0.4)	834 (0.3)	

**Socioeconomic status**			<0.001
1 (least deprived)	56 800 (23.4)	53 034 (21.9)	
2	61 647 (25.4)	60 501 (25.0)	
3	50 466 (20.8)	50 709 (20.9)	
4	42 221 (17.4)	44 692 (18.4)	
5 (most deprived)	31 215 (12.9)	33 413 (13.8)	

**Smoking status**			<0.001
Non-smoker	92 363 (38.1)	80 721 (33.3)	
Ex-smoker	107 737 (44.5)	131 510 (54.3)	
Current smoker	36 338 (15.0)	29 243 (12.1)	
Missing	5911 (2.4)	875 (0.4)	

**Body mass index, kg/m^2^**			<0.001
<18.5	6638 (2.7)	4562 (1.9)	
18.5–25	85 473 (35.3)	70 102 (28.9)	
≥25	80 458 (33.2)	88 083 (36.4)	
≥30	40 326 (16.6)	63 183 (26.1)	
Missing	29 454 (12.2)	16 419 (6.8)	

**Comorbidities**			
Asthma	28 002 (11.6)	31 271 (12.9)	<0.001
Atrial fibrillation	15 448 (6.4)	29 515 (12.2)	<0.001
Cancer	47 431 (19.6)	54 450 (22.5)	<0.001
Chronic obstructive pulmonary disease	14 996 (6.2)	18 229 (7.5)	<0.001
Coronary heart disease	27 961 (11.5)	54 049 (22.3)	<0.001
Dementia	8954 (3.7)	7345 (3.0)	<0.001
Depression	38 490 (15.9)	46 233 (19.1)	<0.001
Diabetes mellitus	24 372 (10.1)	52 927 (21.8)	<0.001
Epilepsy	3972 (1.6)	3682 (1.5)	0.001
Heart failure	7581 (3.1)	23 774 (9.8)	<0.001
Hypertension	128 828 (53.2)	203 963 (84.2)	<0.001
Hypothyroidism	17 443 (7.2)	29 318 (12.1)	<0.001
Severe mental illness	3522 (1.5)	4890 (2.0)	<0.001
Osteoporosis	16 469 (6.8)	16 610 (6.9)	0.422
Peripheral arterial disease	7481 (3.1)	14 815 (6.1)	<0.001
Rheumatoid arthritis	4270 (1.8)	6031 (2.5)	<0.001
Stroke and transient ischaemic attack	12 243 (5.1)	19 982 (8.3)	<0.001

CKD = chronic kidney disease

Among the 10 cause-specific hospitalisations, the largest incidence rate difference was seen for heart failure at 6.6/1000 person-years (9.7/1000 versus 3.1/1000 person-years in matched patients with and without CKD, respectively), followed by urinary tract infection at 5.2/1000 person-years, pneumonia at 4.4/1000 person-years, and AKI at 4.1/1000 person-years (Table 2). Hip fracture, venous thromboembolism, and intracranial bleeding marked relatively small differences of incidence rates between matched patients with and without CKD.

**Table 2.** Difference in the incidence rate of cause-specific hospitalisation between matched patients with and without chronic kidney disease by rank order of rate difference

**Cause of hospitalisation**	**Number of outcome, *n***		**Incidence rate per 1000 person-years (95%CI)**		
	**Patients with CKD (*N*= 242 349)**	**Patients without CKD (*N*= 242 349)**	**Patients with CKD**	**Patients without CKD**	**Rate difference**
Heart failure	10 394	2955	9.7 (9.5 to 9.9)	3.1 (3.0 to 3.2)	6.6 (6.4 to 6.8)
Urinary tract infection	14 266	7654	13.1 (12.9 to 13.3)	7.9 (7.7 to 8.1)	5.2 (4.9 to 5.5)
Pneumonia	13 483	7803	12.6 (12.4 to 12.8)	8.2 (8.0 to 8.4)	4.4 (4.1 to 4.7)
Acute kidney injury	5257	787	4.9 (4.7 to 5.0)	0.8 (0.8 to 0.9)	4.1 (3.9 to 4.2)
Myocardial infarction	7418	3590	6.9 (6.8 to 7.1)	3.8 (3.6 to 3.9)	3.2 (3.0 to 3.4)
Cerebral infarction	6142	3335	5.7 (5.6 to 5.8)	3.5 (3.4 to 3.6)	2.2 (2.0 to 2.4)
Gastrointestinal bleeding	5492	3048	5.1 (5.0 to 5.2)	3.2 (3.1 to 3.3)	1.9 (1.7 to 2.1)
Hip fracture	9336	6751	8.7 (8.6 to 8.9)	7.1 (7.0 to 7.3)	1.6 (1.4 to 1.9)
Venous thromboembolism	3299	1882	3.1 (3.0 to 3.2)	2.0 (1.9 to 2.1)	1.1 (1.0 to 1.2)
Intracranial bleeding	2144	1427	2.0 (1.9 to 2.1)	1.5 (1.4 to 1.6)	0.5 (0.4 to 0.6)

CKD = chronic kidney disease.

The relative risk was consistently highest for AKI, followed by heart failure in all the models, though the rank order of other outcomes varied depending on the extent of adjustment for confounding factors (Table 3). The age- and sex-adjusted hazard ratio for AKI in Model 1 was 6.49, 95% CI = 5.99 to 7.03, followed by heart failure with 3.28, 95% CI = 3.15 to 3.41. The fully adjusted hazard ratio for AKI in Model 4 was 4.90, 95% CI = 4.47 to 5.38, followed by heart failure with 1.66, 95% CI = 1.59 to 1.75. Intracranial bleeding and hip fracture marked relatively small fully adjusted hazard ratios. Results of competing risk analyses were generally similar to or slightly higher than those estimated in the main analysis. Likewise, AKI and heart failure exhibited higher sub-hazard ratios than others.

**Table 3.** Relative risk for cause-specific hospitalisation between matched patients with and without chronic kidney disease by rank order of fully adjusted hazard ratio

**Cause of hospitalisation**	**Adjusted hazard ratio (95% CI)^a^**				**Fully adjusted sub-hazard ratio (95% CI)^b^**
	**Model 1**	**Model 2**	**Model 3**	**Model 4 (fully adjusted model)**	
Acute kidney injury	6.49 (5.99 to 7.03)	5.95 (5.46 to 6.47)	5.82 (5.34 to 6.35)	4.90 (4.47 to 5.38)	4.98 (4.23 to 5.87)
Heart failure	3.28 (3.15 to 3.41)	2.84 (2.73 to 2.96)	2.79 (2.67 to 2.90)	1.66 (1.59 to 1.75)	2.07 (1.88 to 2.28)
Venous thromboembolism	1.60 (1.53 to 1.68)	1.57 (1.49 to 1.65)	1.54 (1.46 to 1.62)	1.55 (1.46 to 1.64)	1.57 (1.37 to 1.80)
Myocardial infarction	1.84 (1.78 to 1.91)	1.70 (1.64 to 1.76)	1.67 (1.61 to 1.73)	1.40 (1.34 to 1.46)	1.53 (1.38 to 1.69)
Urinary tract infection	1.62 (1.58 to 1.67)	1.53 (1.49 to 1.57)	1.50 (1.46 to 1.54)	1.39 (1.35 to 1.43)	1.59 (1.50 to 1.69)
Gastrointestinal bleeding	1.59 (1.53 to 1.66)	1.55 (1.49 to 1.62)	1.52 (1.46 to 1.58)	1.34 (1.28 to 1.40)	1.55 (1.41 to 1.72)
Cerebral infarction	1.55 (1.49 to 1.61)	1.51 (1.46 to 1.58)	1.51 (1.45 to 1.58)	1.27 (1.22 to 1.33)	1.45 (1.30 to 1.60)
Pneumonia	1.47 (1.43 to 1.51)	1.46 (1.42 to 1.50)	1.44 (1.40 to 1.49)	1.24 (1.20 to 1.29)	1.49 (1.39 to 1.59)
Hip fracture	1.11 (1.08 to 1.14)	1.18 (1.14 to 1.21)	1.17 (1.13 to 1.21)	1.11 (1.07 to 1.15)	1.37 (1.27 to 1.48)
Intracranial bleeding	1.28 (1.21 to 1.36)	1.30 (1.22 to 1.38)	1.29 (1.21 to 1.38)	1.10 (1.02 to 1.19)	1.30 (1.11 to 1.52)

a Adjusted hazard ratio (patients with chronic kidney disease versus those without) was estimated in the following Cox regression models: Model 1: Stratified by matched set to account for the matching on age, sex, general practice, and calendar time. Model 2: Model 1 + adjusted by ethnicity, socioeconomic and smoking status, body mass index, and diabetes mellitus. Model 3: Model 2 + adjusted by comorbidities not directly related to chronic kidney disease (asthma, cancer, chronic obstructive pulmonary disease, dementia, depression, epilepsy, hypothyroidism, severe mental illness, osteoporosis, and rheumatoid arthritis). Model 4: Model 3 + adjusted by all the other comorbidities that may occur concordantly with chronic kidney disease (atrial fibrillation, coronary heart disease, heart failure, hypertension, peripheral arterial disease, and stroke and transient ischaemic attack).

b Fully adjusted sub-hazard ratio was estimated by the model of Fine and Gray to account for competing risk (initiation of renal replacement therapy and death) between patients with and without chronic kidney disease, using a 20% random sample of the whole dataset.

In subgroup analysis by age, the incidence rate difference between matched patients with and without CKD tended to be larger and the relative risk tended to be smaller in the older subgroup (≥75 years of age) than the younger subgroup (<75 years of age) for almost all the cause-specific hospitalisations (Table 4). However, the rank order in the size of absolute rate difference and relative risk was almost the same as the main results in each age group.

**Table 4.** Difference in the incidence rate of cause-specific hospitalisation and relative risk between matched patients with and without chronic kidney disease by age

**Causes hospitalisation**	**Patients aged ≥75 years (*N*= 140 836 matched pairs)**				**Patients aged**<**75 years (*N*= 101 513 matched pairs)**			
	**Incidence rate per 1000 person years**			**Fully adjusted HR^a^ (95% CI)**	**Incidence rate per 1000 person-years (95% CI)**			**Fully adjusted HR^a^ (95% CI)**
	**Patients with CKD**	**Patients without CKD**	**Rate difference**		**Patients with CKD**	**Patients without CKD**	**Rate difference**	
Heart failure	13.0 (12.7 to 13.3)	4.8 (4.6 to 5.0)	8.2 (7.9 to 8.6)	1.61 (1.52 to 1.70)	6.0 (5.8 to 6.2)	1.4 (1.3 to 1.5)	4.6 (4.4 to 4.8)	1.78 (1.61 to 1.98)
Urinary tract infection	18.5 (18.2 to 18.9)	12.8 (12.5 to 13.1)	5.7 (5.2 to 6.2)	1.27 (1.23 to 1.32)	7.8 (7.6 to 8.0)	3.5 (3.3 to 3.6)	4.3 (4.0 to 4.6)	1.71 (1.61 to 1.81)
Pneumonia	17.4 (17.1 to 17.8)	12.6 (12.2 to 12.9)	4.9 (4.4 to 5.3)	1.17 (1.13 to 1.22)	7.2 (7.0 to 7.4)	3.9 (3.8 to 4.1)	3.3 (3.0 to 3.6)	1.46 (1.36 to 1.57)
Acute kidney injury	5.5 (5.3 to 5.7)	1.2 (1.1 to 1.3)	4.3 (4.1 to 4.5)	4.27 (3.80 to 4.80)	4.2 (4.0 to 4.4)	0.5 (0.4 to 0.5)	3.7 (3.5 to 3.9)	6.64 (5.62 to 7.84)
Myocardial infarction	8.8 (8.6 to 9.1)	5.0 (4.8 to 5.2)	3.9 (3.6 to 4.2)	1.43 (1.35 to 1.50)	4.8 (4.6 to 5.0)	2.6 (2.4 to 2.7)	2.2 (1.9 to 2.4)	1.34 (1.24 to 1.44)
Cerebral infarction	7.8 (7.6 to 8.1)	5.3 (5.1 to 5.5)	2.6 (2.3 to 2.9)	1.22 (1.16 to 1.29)	3.3 (3.2 to 3.5)	1.8 (1.6 to 1.9)	1.6 (1.4 to 1.8)	1.42 (1.29 to 1.56)
Gastrointestinal bleeding	6.3 (6.1 to 6.5)	4.2 (4.0 to 4.4)	2.1 (1.9 to 2.4)	1.34 (1.27 to 1.42)	3.7 (3.6 to 3.9)	2.2 (2.1 to 2.3)	1.5 (1.3 to 1.7)	1.31 (1.21 to 1.42)
Hip fracture	13.8 (13.5 to 14.1)	12.3 (12.0 to 12.6)	1.6 (1.1 to 2.0)	1.07 (1.03 to 1.11)	3.1 (2.9 to 3.2)	2.1 (2.0 to 2.3)	0.9 (0.7 to 1.1)	1.31 (1.19 to 1.43)
Venous thromboembolism	3.4 (3.2 to 3.5)	2.4 (2.3 to 2.6)	1.0 (0.8 to 1.2)	1.48 (1.37 to 1.60)	2.7 (2.6 to 2.8)	1.5 (1.4 to 1.6)	1.2 (1.0 to 1.4)	1.65 (1.50 to 1.81)
Intracranial bleeding	2.7 (2.5 to 2.8)	2.2 (2.1 to 2.3)	0.5 (0.3 to 0.7)	1.06 (0.97 to 1.16)	1.2 (1.1 to 1.3)	0.8 (0.7 to 0.9)	0.4 (0.3 to 0.5)	1.19 (1.03 to 1.37)

a Fully adjusted hazard ratio (patients with versus without CKD) was estimated using Cox regression models, stratified by matched set to account for the matching on age, sex, general practice, and calendar time, and adjusted by ethnicity, socioeconomic and smoking status, body mass index, and comorbidities (asthma, atrial fibrillation, cancer, chronic obstructive pulmonary disease, coronary heart disease, dementia, depression, diabetes mellitus, epilepsy, heart failure, hypertension, hypothyroidism, severe mental illness, osteoporosis, peripheral arterial disease, rheumatoid arthritis, and stroke and transient ischaemic attack). CKD = chronic kidney disease. HR = hazard ratio.

**Figure 2.**
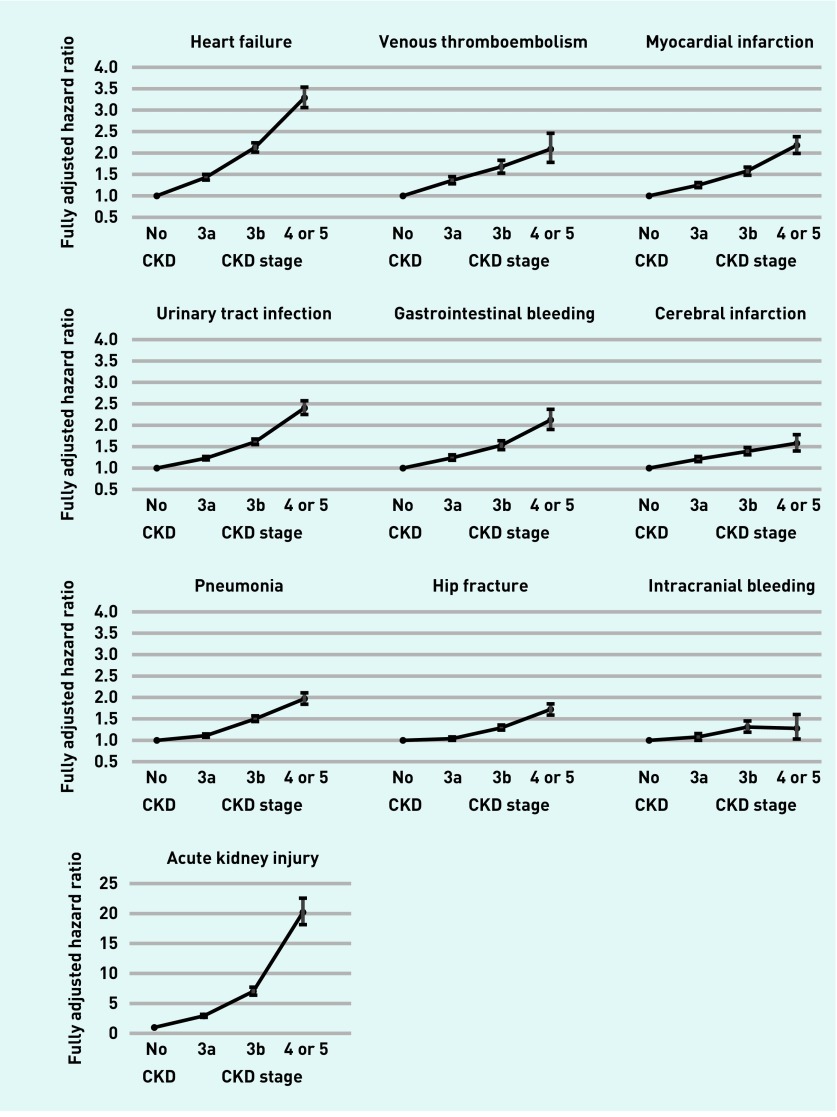
***Fully adjusted hazard ratio for cause-specific hospitalisation by chronic kidney disease stage estimated using Cox regression models, adjusted by age, sex, financial year, ethnicity, socioeconomic and smoking status, body mass index, and comorbidities (asthma, atrial fibrillation, cancer, chronic obstructive pulmonary disease, coronary heart disease, dementia, depression, diabetes mellitus, epilepsy, heart failure, hypertension, hypothyroidism, severe mental illness, osteoporosis, peripheral arterial disease, rheumatoid arthritis, and stroke and transient ischaemic attack) and clustered by general practice using robust standard errors (corresponding to Appendix 2).*** ***CKD = chronic kidney disease.***

Of 242 349 matched patients with CKD, 71.2% (*n* = 172 555), 22.9% (*n* = 55 500), and 5.9%, (*n* = 14 294) patients were in stage 3a, 3b, and 4 or 5, respectively. Patients tended to be older and sicker as kidney function declined. Details of baseline characteristics of patients with different stages of CKD are available from the authors. There were graded associations between CKD stage and all the cause-specific hospitalisations, but the strength of the association was larger for AKI and heart failure (Figure 2 and Appendix 2).

Among patients with no history of cardiovascular disease, the strength of the association was similar to that in the main analysis for all the studied cause-specific hospitalisations (Appendix 3).

## DISCUSSION

### Summary

In this population-based cohort study, among people with CKD (stages 3–5) large absolute increases in rates of hospitalisations due to heart failure, infection (urinary tract infection and pneumonia), and AKI were found, compared with age- and sex-matched controls without known CKD from the same GP. Before and after adjustment for confounding factors, the relative risk of hospitalisation was highest for AKI, followed by heart failure. Results were similar in subgroup analyses by age and CKD stage, and among patients with no history of cardiovascular disease. The vast majority of patients in the cohort had CKD stage 3a or 3b so would be primarily diagnosed and managed in primary care, making these findings useful and relevant for routine clinical care.

### Strengths and limitations

A major strength of this study was that it compared those with CKD to those without, sampled from the general population. A comparison was possible because over 98% of the UK population are registered with a primary care practice. The study results obtained from HES-linked CPRD are likely to be generalisable to the entire English population.^29^

Limitations of the study include, first, that patients who had never had kidney function tested were kept in the denominator in order for the comparison group to be representative of the general population. Currently in the UK, serum creatinine testing is recommended and incentivised for people with known CKD risk factors.^3^^,^^41^ If some patients had been misclassified with unmeasured CKD to the matched comparison group, the true association between CKD and each cause-specific hospitalisation may have been underestimated. However, the researchers have recently shown that the prevalence of patients with an eGFR <60 mL/min/1.73 m^2^ identified in CPRD was similar to that in a population-representative survey (Health Survey for England),^42^ suggesting that most of these patients are captured with the current testing strategy in UK primary care, and people without creatinine tests are unlikely to have CKD stages 3–5.^43^ If healthy people without creatinine measurement were excluded from the denominator, severe selection bias would arise and estimated absolute risk differences between patients with and without CKD would not be informative.^44^

Second, relative risks between CKD status (stage) and cause-specific hospitalisation depend partly on the extent of adjustment for potential confounders. Previous studies on the association between CKD and outcomes adjusted for disease diagnoses (based on patient charts, administrative claim data, or questionnaire answered by patients), physiological measurements, blood test results, or prescriptions to various degrees conclude that CKD is ‘independently’ associated with their studied outcomes.^15^^–^28 However, the possibility of residual confounding inevitably remains. In this study, adjustments were made for important patient characteristics as well as diagnoses of 17 comorbidities. Recording of these conditions has been incentivised since the introduction of the UK Quality and Outcomes Framework in 2004,^3^ resulting in marked improvements in data quality.^29^ Further, differences of disease diagnosis and coding among different GPs and over time was minimised by matching on GP and calendar time between patients with and without CKD. Therefore, the authors believe that the best available approach of adjustment for confounding was used to compare the relative risks between CKD and different types of cause-specific hospitalisations.

Finally, although the researchers acknowledge that proteinuria is an important outcome prediction marker,^32^ the authors of this study were not able to stratify patients by the level of proteinuria or quantify the association between proteinuria and cause-specific hospitalisation. This is because proteinuria was infrequently checked in CPRD: testing rate of proteinuria (including dipstick testing) in the year prior to cohort entry was 23.2% (56 431 out of 242 349 patients) in the CKD group and 12.6% (30 616 out of 242 349 patients) in the comparison group. It would not be appropriate to assume that people without urine testing did not have proteinuria.

### Comparison with existing literature

There have been several studies demonstrating an association between CKD and increased risk of all-cause hospitalisations.^9^^,^^11^^–^13 However, these studies did not clearly differentiate causes of hospitalisation, and therefore it remained unclear why this is the case. Meanwhile, a previous study recruiting patients with elevated serum creatinine suggested that cardiovascular disease and hypertension were the most common reason for hospitalisation, followed by infection.^45^ However, in the absence of a comparison group without CKD, it remained unclear whether these hospitalisations were specific to CKD or common in the community regardless of CKD status.

As well as a known association between CKD and AKI,^46^ a number of studies have reported positive associations between CKD and incidence of non-renal conditions.^15^^–^28 Many of these studies used hospital admission for their outcome definitions. However, these individual studies did not allow a comparison of the impact of CKD across different outcomes, because absolute and relative risk related to CKD were estimated in different study populations and with various degrees of statistical adjustment for confounders. To the authors’ knowledge, the current study is the first to quantify the association between CKD status (stage) and cause-specific hospitalisation.

### Implications for research and practice

After the classification of CKD, and the implementation of testing and registering of patients with CKD through the Quality and Outcomes Framework for CKD in 2006,^3^ some have questioned the benefits of this approach.^47^ Patients with mild CKD may be perceived to have normal kidney ageing, or with multiple morbidities putting them at increased risk for many adverse outcomes. This study was planned to clarify the adverse outcomes (that were likely to be causally related to kidney function) that were more common and specific among patients with CKD in primary care, enabling the possibility of better-targeted care.

The adjusted hazard ratios were small for most of the outcomes in patients with CKD stage 3a (Figure 2), except for AKI with a threefold increase in the adjusted hazard ratio for hospitalisations; and nearly one and a half-fold increase for heart failure. These results highlight the marked increase in risk of AKI and heart failure for patients with only mild reductions in kidney function, with implications for targeted prevention and medication management; for example, minimisation of non-steroidal antiinflammatory drug use.

Both absolute and relative risk provide important information about the impact of CKD on cause-specific hospitalisation.^40^^,^^48^ The relative risk (adjusted hazard ratio) is a measure of the strength of the association between CKD and each cause-specific hospitalisation, after taking into account a range of comorbidities. Meanwhile, the absolute risk difference reflects the relative risk and the baseline frequency of each outcome in the community, indicating the actual burden of each condition among patients with CKD as compared with the general population. For example, infections, such as urinary tract infection and pneumonia, showed intermediate relative risks among the studied outcomes, but their absolute risk differences between patients with and without CKD were large because hospitalisations for infection were common in the general population. The absolute risk difference is also useful for understanding the potential benefits of preventive strategies. For example, Table 2 shows that 9.7 and 3.1 patients per 1000 patients with and without CKD (stages 3–5), respectively, were hospitalised for heart failure in a year, meaning that, of 1000 people with CKD, up to 6.6 could benefit from targeted heart failure admission prevention. This would translate to three people per year in a GP practice of 7400 patients (average number of patients per practice)^49^ where 6.5% have CKD. Further estimates for all outcomes are available from the authors. However, these numbers are likely to be underestimates of the overall benefits because follow-up of patients at the time of first hospitalisation after cohort entry was stopped and, therefore, did not account for repeated admissions.

Patients with CKD, even without renal replacement therapy, are known to incur substantive healthcare costs through frequent hospitalisations.^10^ Based on results from this study, a focus on strategies to reduce hospitalisations for heart failure, such as education on dietary salt restriction^50^ and improved medication adherence,^51^ could help to minimise the difference in the overall hospitalisation rate between patients with and without CKD. Similarly, a proportion of hospitalisations due to infections may be preventable through prompt antibiotic treatment and improvement of vaccination coverage among patients with CKD.^52^ Pneumococcal vaccination has been underutilised in patients with CKD (stages 4 and 5) to date.^53^

## Appendix 1. List of International Classification of Diseases 10th Revision codes used to identify cause-specific hospitalisations

**Table:**

**Outcome**	**International Classification of Diseases 10th Revision codes**
Myocardial infarction	I21, I22, I23
Heart failure	I50
Cerebral infarction	I63
Pneumonia	B01.2, B05.2, B20.6, B25.0, J10.0, J11.0, J12, J13, J14, J15, J16, J17, J18, J85.1, U04
Urinary tract infection	N10, N12, N13.6, N15.1, N15.9, N30.0, N30.8, N30.9, N39.0
Gastrointestinal bleeding	I85.0, K22.6, K25.0, K25.1, K25.2, K25.4, K25.5, K25.6, K26.0, K26.1, K26.2, K26.4, K26.5, K26.6, K27.0, K27.1, K27.2, K27.4, K27.5, K27.6, K28.0, K28.1, K28.2, K28.4, K28.5, K28.6, K29.0, K92.0, K92.1, K92.2
Intracranial bleeding	I60, I61, I62, S06.5, S06.6
Venous thromboembolism	I80.1, I80.2, I80.3, I81, I82.2, I82.3, I82.8, I82.9, I26
Hip fracture	S72
Acute kidney injury	N17

## Appendix 2. Fully adjusted hazard ratio for cause-specific hospitalisation by chronic kidney disease stage

**Table:**

**Cause of hospitalisation**	**Fully adjusted hazard ratio (95% CI)^a^ by CKD stage**			
	**Patients without CKD (*N*= 242 349)**	**Patients with CKD (*N*= 242 349)**		
		**CKD stage 3a (*N*= 172 555)**	**CKD stage 3b (*N*= 55 500)**	**CKD stage 4 or 5 (*N*= 14 294)**
Acute kidney injury	1^b^	2.94 (2.69 to 3.20)	7.03 (6.39 to 7.73)	20.22 (18.14 to 22.54)
Heart failure	1^b^	1.43 (1.37 to 1.50)	2.13 (2.02 to 2.24)	3.29 (3.06 to 3.54)
Venous thromboembolism	1^b^	1.36 (1.28 to 1.45)	1.68 (1.53 to 1.83)	2.09 (1.78 to 2.46)
Myocardial infarction	1^b^	1.25 (1.19 to 1.31)	1.58 (1.48 to 1.67)	2.18 (1.99 to 2.38)
Urinary tract infection	1^b^	1.23 (1.19 to 1.27)	1.61 (1.55 to 1.68)	2.40 (2.25 to 2.57)
Gastrointestinal bleeding	1^b^	1.24 (1.18 to 1.31)	1.53 (1.43 to 1.64)	2.12 (1.90 to 2.37)
Cerebral infarction	1^b^	1.21 (1.15 to 1.27)	1.39 (1.31 to 1.48)	1.58 (1.40 to 1.78)
Pneumonia	1^b^	1.11 (1.07 to 1.15)	1.50 (1.44 to 1.57)	1.97 (1.84 to 2.11)
Hip fracture	1^b^	1.04 (1.00 to 1.08)	1.29 (1.24 to 1.36)	1.72 (1.59 to 1.85)
Intracranial bleeding	1^b^	1.08 (1.00 to 1.16)	1.31 (1.19 to 1.45)	1.28 (1.03 to 1.60)

a Fully adjusted hazard ratio was estimated using Cox regression models, adjusted by age, sex, financial year, ethnicity, socioeconomic and smoking status, body mass index, and comorbidities (asthma, atrial fibrillation, cancer, chronic obstructive pulmonary disease, coronary heart disease, dementia, depression, diabetes mellitus, epilepsy, heart failure, hypertension, hypothyroidism, severe mental illness, osteoporosis, peripheral arterial disease, rheumatoid arthritis, and stroke and transient ischaemic attack) and clustered by general practice using robust standard errors.

b Reference. CKD = chronic kidney disease.

## Appendix 3. Fully adjusted hazard ratio for cause-specific hospitalisation by chronic kidney disease stage among patients with no history of cardiovascular disease

**Table:**

**Cause of hospitalisation**	**Fully adjusted hazard ratio (95% CI)^a^ by CKD stage**			
	**Patients without CKD (*N* = 187 322)**	**Patients with CKD (*N*= 143 715)**		
		**CKD stage 3a (*N*= 107 803)**	**CKD stage 3b (*N*= 29 085)**	**CKD stage 4 or 5 (*N*= 6827)**
Acute kidney injury	1^b^	2.65 (2.39 to 2.93)	7.09 (6.29 to 8.00)	24.20 (21.22 to 27.61)
Heart failure	1^b^	1.25 (1.17 to 1.35)	1.95 (1.78 to 2.14)	3.41 (2.96 to 3.94)
Venous thromboembolism	1^b^	1.43 (1.33 to 1.54)	1.66 (1.48 to 1.85)	2.23 (1.80 to 2.76)
Myocardial infarction	1^b^	1.25 (1.16 to 1.34)	1.61 (1.47 to 1.76)	2.24 (1.92 to 2.60)
Urinary tract infection	1^b^	1.27 (1.21 to 1.33)	1.75 (1.66 to 1.85)	3.15 (2.87 to 3.46)
Gastrointestinal bleeding	1^b^	1.29 (1.20 to 1.38)	1.56 (1.41 to 1.72)	2.13 (1.79 to 2.54)
Cerebral infarction	1^b^	1.19 (1.11 to 1.28)	1.45 (1.33 to 1.58)	1.56 (1.31 to 1.86)
Pneumonia	1^b^	1.14 (1.09 to 1.19)	1.56 (1.47 to 1.65)	2.33 (2.11 to 2.58)
Hip fracture	1^b^	1.03 (0.98 to 1.08)	1.27 (1.19 to 1.35)	1.69 (1.50 to 1.91)
Intracranial bleeding	1^b^	1.10 (0.99 to 1.21)	1.42 (1.24 to 1.63)	1.35 (0.98 to 1.85)

a After excluding patients with a history of cardiovascular disease (atrial fibrillation, coronary heart disease, heart failure, peripheral arterial disease, or stroke and transient ischaemic attack) from the matched patients with and without CKD, fully adjusted hazard ratios were estimated using Cox regression models, adjusted by age, sex, financial year, ethnicity, socioeconomic and smoking status, body mass index, and comorbidities (asthma, cancer, chronic obstructive pulmonary disease, dementia, depression, diabetes mellitus, epilepsy, hypertension, hypothyroidism, severe mental illness, osteoporosis, and rheumatoid arthritis) and clustered by general practice using robust standard errors.

b Reference. CKD = chronic kidney disease.
